# Intracranial efficacy and safety of furmonertinib 160 mg with or without anti-angiogenic agent in advanced NSCLC patients with BM/LM as salvage therapy

**DOI:** 10.1186/s12885-023-10676-x

**Published:** 2023-03-04

**Authors:** Ziyi Xu, Xuezhi Hao, Qi Wang, Ke Yang, Junling Li, Puyuan Xing

**Affiliations:** 1 Department of Medical Oncology, National Cancer Center/National Clinical Research Center for Cancer/Cancer Hospital, Chinese Academy of Medical Sciences and Peking Union Medical College, Beijing, 100021 China; 2 Department of Medical Oncology, Beijing Chaoyang Sanhuan Hospital, Beijing, 100021 China; 3 Department of Medical Oncology, Cancer Hospital of Huanxing, Beijing, 100021 China

**Keywords:** *EGFR*-mutated NSCLC, BM/LM, Furmonertinib, Anti-angiogenic agent, Salvage therapy

## Abstract

**Objectives:**

Central nervous system (CNS) metastases including brain metastases (BM) and leptomeningeal metastases (LM) are frequent in epidermal growth factor receptor (*EGFR*)-mutated non-small cell lung cancer (NSCLC), and are correlated with poor outcomes. In this study, we evaluated the efficacy of single-agent furmonertinib 160 mg or combining with anti-angiogenic agent in NSCLC patients who had developed BM/LM progression from previous tyrosine kinase inhibior (TKI) treatment.

**Methods:**

*EGFR*-mutated NSCLC patients who developed BM (the BM cohort) or LM progression (the LM cohort) were included, having received furmonertinib 160 mg daily as second-line or later treatment, with or without anti-angiogenic agents. The intracranial efficacy was evaluated by intracranial progression-free survival (iPFS).

**Results:**

Totally 12 patients in the BM cohort and 16 patients in the LM cohort were included. Almost one half of patients in the BM cohort and a majority in the LM cohort had a poor physical status, with a Eastern Cooperative Oncology Group performance status (ECOG-PS) ≥2. The administration of single-agent furmonertinib or combination treatment achieved a median iPFS of 3.6 months (95%CI 1.435–5.705) in the BM cohort, and 4.3 months (95%CI 2.094–6.486) in the LM cohort. Subgroup and univariate analysis has shown that a good ECOG-PS correlated with a favorable efficacy of furmonertinib in the BM cohort (median iPFS = 2.1 with ECOG-PS ≥ 2 vs. 14.6 months with ECOG-PS < 2, *P* < 0.05). Overall, any grade of adverse events (AEs) occured in 46.4% of patients (13/28). Among them, 14.3% of patients (4 of 28) had grade 3 or higher AEs, and were all under control, led to no dose reductions or suspension.

**Conclusion:**

Single-agent furmonertinib 160 mg or in combination of anti-angiogenic agent is an optional salvage therapy for advanced NSCLC patients who developed BM/LM progression from prior *EGFR*-TKI treatment, with a promising efficacy and an acceptable safety profile, and is worth of further exploration.

**Supplementary Information:**

The online version contains supplementary material available at 10.1186/s12885-023-10676-x.

## Introduction

Lung cancer is one of the leading cause of cancer-related deaths worldwide [1], and non-small cell lung cancer (NSCLC) accounts for approximately 85% of lung cancer. The development of tyrosine kinase inhibior (TKI) has greatly altered the standard of care for advanced NSCLC with driver gene mutations, for example, epidermal growth factor receptor (*EGFR*) sensitive mutation [2] and anaplastic lymphoma kinase (*ALK*) mutation [3]. Multiple trials have demonstrated the efficacy of *EGFR-*TKIs in *EGFR*-mutated NSCLC patients in promoting response and prolonging survival compared to standard chemotherapy [4, 5]. Third-generation TKIs such as osimertinib and aumolertinib are selective for both *EGFR* sensitive mutation [6, 7] and *EGFR* T790M resistant mutation [8, 9], which occurred in probably 50% of patients who developed resistance to first- and second-generation TKIs [10].

The frequency of brain metastases (BM) is reported to be 20% at diagnosis and 25–50% during the course of NSCLC [11], and to be 48–50% in patients with *EGFR* sensitizing mutations [12]. BM is not only associated with poor outcome, but also leads to the impairment of quality of life due to the neurological symptoms thus caused. Leptomeningeal metastases (LM) is another central nervous system (CNS) disease that occurs in 3–4% of NSCLC patients, and in approximate 9% of those with *EGFR* mutations [13]. Patients diagnosed as LM have a median overall survival (OS) of 3–10 months [14, 15] with limited therapeutic options that are effective. As characterized as the spread of tumor cells into the leptomeninges and cerebral spinal fluid (CSF), the intracranial concentration of drugs may correlate with their efficacy in LM. Therefore, there is an urgent clinical need for agents with improved blood–brain barrier (BBB) penetration and improvement in CNS disease control. Third-generation *EGFR*-TKI osimertinib has shown a superior CNS activity compared to other TKIs and cytotoxic agents [16, 17].

Furmonertinib is a newly-developed irreversible third-generation *EGFR*-TKI with a trifluoroethoxypyridine-based molecule structure [18]. In the dose escalation study, the objective response rate (ORR) of furmonertinib for T790M-positive NSCLC patients was 66.7 and 66.7% in the 80 mg and 160 mg group, respectively, with no dose-limiting toxicity (DLT) observed [18]. The dose-expansion study has indicated the preliminary efficacy of furmonertinib 80 mg in NSCLC patients harboring *EGFR* T790M mutation, with the ORR of 77.8% and the median progression-free survial (mPFS) of 11.1 months in the 80 mg group [18]. In the phase 2b study which assessed the efficacy and safety of furmonertinib in patients with *EGFR* T790M mutated advanced NSCLC, the ORR was 74%, and the mPFS was 9.6 months (95% CI 8.2–9.7) [19]. In the phase 3 FURLONG study, furmonertinib showed superior efficacy over gefitinib in PFS as first-line therapy in *EGFR*-mutated NSCLC patients (20.8 versus 11.1 months, HR = 0.44, 95%CI 0.34–0.58, *p* < 0.0001) [20]. Although the dose of 80 mg was recommended in consideration of efficacy and safety comprehensively, the dose of 160 mg has also shown a promising efficacy and an acceptable safety profile in the phase 2 study, especially in those with CNS metastases [18]. However, the real-world evidence of double-dose furmonertinib is still lacking. As a good Eastern Cooperative Oncology Group (ECOG) performance status (PS) and a naive history of treatment was required for inclusion in most perspective randomized clinical trials nowadays, the efficacy and tolerability of double-dose furmonertinib in patients who were heavily-treated and physically-weak still requires to be explored in the real world. Therefore, we designed this study to explore the efficacy and safety of single agent furmonertinib or combining with anti-angiogenic agent in advanced NSCLC patients who failed previous TKIs and progressed in BM/LM in the real-world setting. In attempts to increase the CNS concentration of furmonertinib to enhance CNS disease control, the dose of 160 mg was applied in this study.

## Methods

### Participants and study design

Patients diagnosed with advanced *EGFR*-mutated NSCLC who developed CNS progression after *EGFR*-TKI therapy were included from Chinese Academy of Medical Sciences (CAMS), during June, 2021 and June, 2022, having received furmonertinib 160 mg daily as second-line or later treatment. Patients included in this study were classified into two cohorts according to the metastatic pattern to prior TKI: the BM cohort included patients who experienced BM progression with or without extracranial progression from prior systemic treatments, without LM, and the LM cohort included patients who developed LM progression following prior TKIs, with or without BM or extracranial progression. LM was confirmed by CSF cytology via lumbar puncture. Generally, a genetic testing via tissue or ctDNA or CSF was recommend to explore the resistant mechanisms after prior TKI, using the next-generation sequencing (NGS) panel that included *EGFR* sensitizing/resistant mutations, *EGFR* amplifications, and other mutations.

The clinicopathological features have been collected from medical records, including their gender, age, *EGFR* mutation status at diagnosis, and clinical stage at diagnosis. Physical condition before the administration of furmonertinib was also recorded, assessed by ECOG-PS. Treatment information including whether other third-generation TKI had been administered prior to furmonertinib (rechallenge), previous lines of systemic therapy, and localized therapy such as radiotherapy and surgery was obtained from records. The dates of furmonertinib initiation and Response Evaluation Criteria of Solid Tumors (RECIST)-defined PD were also obtained.

### Assessments of efficacy and safety

The assessment of efficacy in patients was done per RECIST version 1.1. PFS was defined as the period from initiation of furmonertinib 160 mg treatment to progression or death from any cause. The intracranial PFS (iPFS) was defined as the time from the initiation of furmonertinib 160 mg to CNS progression or death of any reason, whichever came first. OS was defined as the period from initiation of furmonertinib 160 mg treatment to death from any cause. CNS ORR in the BM cohort was defined as the proportion of patients with a complete response (CR) or partial response (PR) in CNS lesions with at least one measurable site. CNS DCR was defined as the percentage of patients with a CNS response of CR or PR or stable disease (SD) in CNS lesions.

General safety analysis was done using the National Cancer Institute-Common Terminology Criteria for Adverse Events (NCI-CTCAE) version 5.0. The CNS-related symptoms were mainly based on the subjective reports from patients. The extent of improvement in CNS-related symptoms after furmonertinib was mainly based on the subjective report from patients, which could be categorized into three different levels (improvement, no improvement, and deterioration).

### Statistical analysis

Statistical analysis was conducted using the SPSS 26.0 statistical software (SPSS, Inc., Chicago, IL, USA). The survival curves were estimated using the Kaplan-Meier method, while differences in the variables were calculated using the log-rank test. A two-sided *p* value < 0.5 was considered statistically significant. Risk factors for iPFS were analyzed in each cohort with the univariate Cox proportional hazards regression model, using the covariates such as age, gender, ECOG-PS, baseline *EGFR* status, progressive pattern, and treatment strategies.

## Results

### Characteristics

A total of 28 advanced *EGFR*-mutated NSCLC patients received furmonertinib 160 mg with or without anti-angiogenic agents after CNS progression to at least one line of systemic treatment. There were 12 patients in the BM cohort and 16 patients in the LM cohort. The baseline demographics were shown in Table 1. In the BM cohort, the median age was 60 (IQR 52–72), and 41.7% of included patients were female. There were 58.3% of patients who were initially diagnosed as stage IV disease. The ECOG-PS in the BM cohort ranged from 0 to 3, and 41.7% of patients had an ECOG-PS of 2–3 before the administration of furmonertinib. In the LM cohort, the median age was 58 (IQR 53–64), and most of the patients were female (62.5%). The majority of patients in the LM cohort had an ECOG-PS of 2–3 (87.5%), and over a half were initially diagnosed as stage IV disease (56.3%), similar to the BM cohort. In the LM cohort, a great number of patients (14/16, 87.5%) had BM lesions in the course of disease, either in early course of treatments, or concurrently with LM progression.

**Table 1:** Clinicopathological characteristics at baseline and treatment strategies

	BM cohort (*N* = 12) N(%)	LM cohort (*N* = 16) N(%)
Age		
Median (IQR)	60 (52–72)	58 (53–62)
Gender		
Female	5 (41.7)	10 (62.5)
Male	7 (58.3)	6 (37.5)
ECOG-PS		
0–1	7 (58.3)	2 (12.5)
2–3	5 (41.7)	14 (87.5)
Clinical stage at diagnosis		
IV	7 (58.3)	9 (56.3)
I-III	5 (41.7)	7 (43.8)
*EGFR* status at baseline		
Exon 19del	5 (41.7)	7 (43.8)
Exon21 L858R	7 (58.3)	6 (37.5)
Other *EGFR* mutations	0 (0.0)	3 (18.8)
*EGFR* status in CSF		
*EGFR* mutations available	–	4 (25.0)
Negative	–	1 (6.3)
Unknown	–	11 (68.8)
CNS-related symptoms		
Presence	3 (25.0)	15 (93.8)
Absence	9 (75.0)	1 (6.3)
*EGFR* status prior to furmonertinib		
Unknown/negative	7 (58.3)	10 (62.5)
T790M mutations	3 (25.0)	1 (6.3)
*EGFR* sensitive mutations	2 (16.7)	5 (31.3)
Previous lines of systemic therapy		
0–1	4 (33.3)	11 (68.8)
2–3	8 (66.7)	5 (31.3)
Rechallenge of 3rd generation TKI		
Yes	9 (75.0)	10 (62.5)
No	3 (25.0)	6 (37.5)
Treatment between 3rd generation TKI and furmonertinib		
Other TKI	1 (11.1)	2 (20.0)
Chemotherapy	5 (55.6)	3 (30.0)
No treatment	3 (33.3)	5 (50.0)
Pre-treated/concurrent with RT		
Yes	10 (83.3)	6 (37.5)
No	2 (16.7)	10 (62.5)
Treatment strategies		
Furmonertinib monotherapy	6 (50.0)	11 (68.8)
Furmonertinib+anti-angiogenic agent	6 (50.0)	5 (31.3)
Intrathecal injection		
Yes	–	9 (56.3)
No	–	7 (43.8)
Regimens for intrathecal injection		
Pemetrexed	–	5 (55.6)
MTX	–	4 (44.4)

The percentages might not equal 100% on account of rounding

*n* number, *ECOG PS* Eastern Cooperative Oncology Group performance status, *EGFR* epidermal growth factor receptor, *CSF* cerebral spinal fluid, *CNS* central nervous system, *TKI* tyrosine kinase inhibior, *RT* radiotherapy

With regard to the gene mutation status, the percentage of patients harboring *EGFR* exon19 deletion and exon21 L858R at baseline was 41.7 and 58.3%, respectively, in the BM cohort, and was 43.8 and 37.5%, respectively, in the LM cohort, with other 3 patients (18.8%) who had other *EGFR* sensitizing mutations at diagnosis such as *EGFR* exon18 mutation. Over a half of patients had unknown or negative results of *EGFR* mutations before the administration furmonertinib in each group, and only 25.0% in the BM cohort and 6.3% in the LM cohort harbored the T790M mutation, via NGS tests in ctDNA. The other 16.7% in the BM cohort and 51.3% in the LM cohort had other *EGFR* sensitive mutations with or without *EGFR* amplifications prior to furmonertinib. In the LM cohort, there were also 43.8% of patients (7/16) received furmonertinib treatment without genetic tests, considering the severity of progressive disease. Only 11.1% of the 9 patients with gene test had T790M mutation before the administration of furmonertinib, over a half of the patients (5/9, 55.6%) harbored other *EGFR* mutations or amplifications, and 33.3% of patients (3/9) had a negative result in *EGFR* detection. NGS was also performed in CSF in 5 patients with LM, whereas no T790M mutation has been detected.

### Treatment history and strategy

The treatment history of the included patients was shown in Table 1. The median number of lines of previous systemic treatments were 3 lines prior to furmonertinib treatment, including targeted therapy and chemotherapy. The majority of patients (67.9%, 19 of 28) had received other third-generation *EGFR*-TKI previously, mostly osimertinib. These patients received furmonertinib as a rechallenge of third-generation TKI, regardless of T790M mutation. There were 33.3% (3/9) and 50.0% (5/10) of patients who switched from prior third-generation TKI to furmonertinib directly in the BM and LM cohort, respectively, while the other 11.1% (1/9) and 55.6% (5/9) of patients who had received other TKI or chemotherapy between prior third-generation TKI to furmonertinib respectively, in the BM cohort, and other 20.0% (2/10) and 30.0% (3/10) respectively, in the LM cohort.

In the BM cohort, 66.7% of patients (8/12) had received more than 1 line of systemic treatments prior to furmonertinib. The majority of patients (10/12, 83.3%) had received radiotherapy (RT) in CNS, with 60.0% (6/10) who had RT in prior treatment, and another 40.0% (4/10) who had RT concurrently with furmonertinib. With regard to the treatment strategy after BM progression, a half of the patients received furmonertinib 160 mg as monotherapy, while the other half received furmonertinib plus anti-angiogenic agent including anlotinib and bevacizumab as subsequent therapy.

In the LM cohort, 68.8% of the patients (11/16) received furmonertinib as second-line therapy. There were 68.8% of patients (11/16) who received single-agent furmonertinib 160, and 56.3% of patients (9/16) who also received intrathecal injection of pemetrexed (55.6%, 5/9) or MTX (44.4%, 4/9). Totally 6 patients (37.5%) had received early local therapies targeting BM lesions before (50.0%, 3/6) or in conjunction with furmonertinib treatment (50.0%, 3/6) in the LM cohort, such as gamma knife therapy and Helical TomoTherapy (TOMO).

### Efficacy of Furmonertinib as salvage treatment

**Fig. 1:**
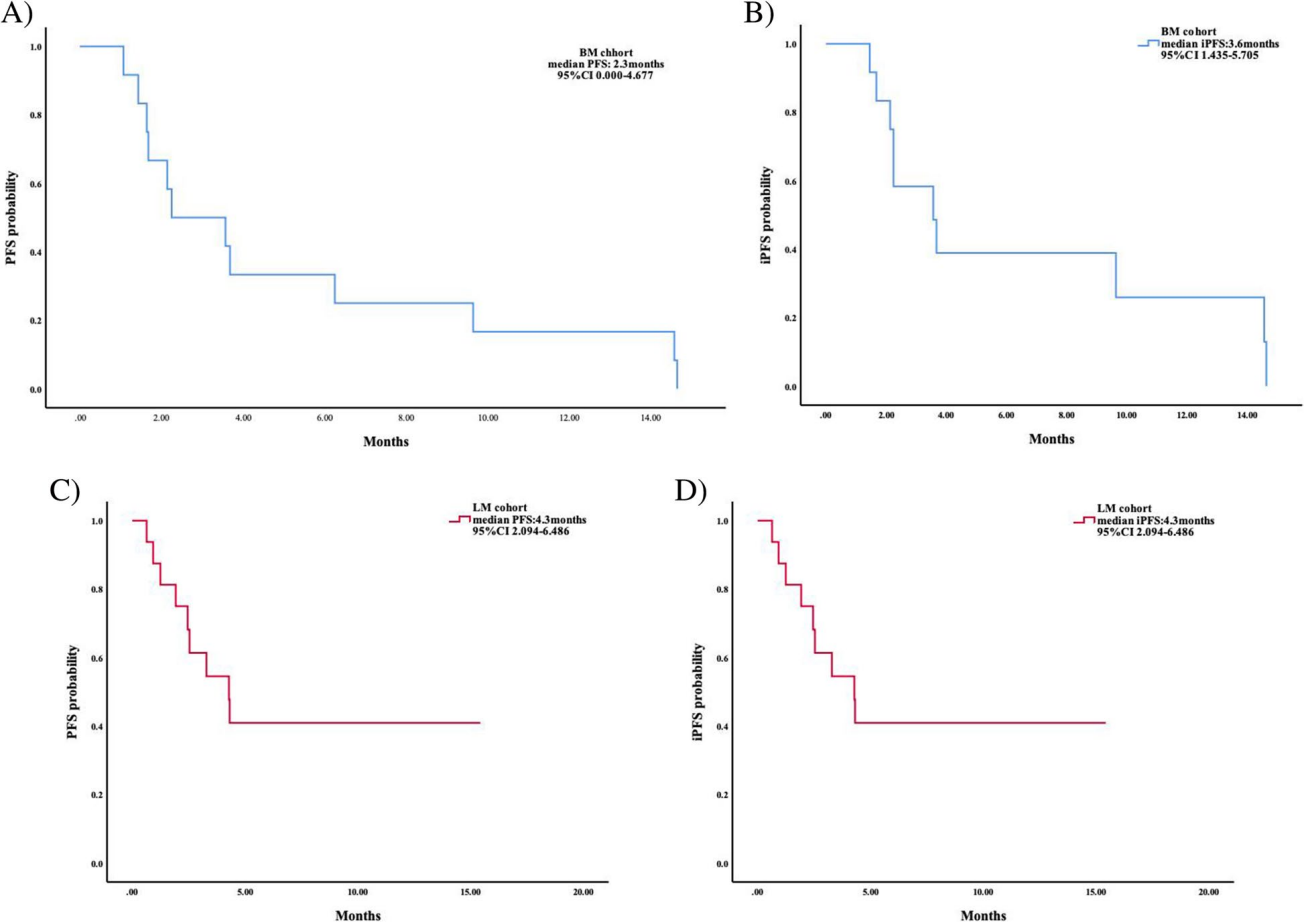
Survival analysis in the brain metastases (BM) and leptomeningeal metastases (LM) cohorts. **A** Median progression-free survival (PFS) and **B** intracranial PFS (iPFS) in the BM cohort. **C** Median PFS and median iPFS (**D**) in the LM cohort

Until the cut-off date of Sept. 16th, 2022, the median follow-up duration in all included patients was 6.3 months (ranging from 1.5–15.1 months), and 7.8 months (ranging from 1.9–15.4 months), respectively in the BM and the LM cohort. OS event occurred in 33.3 and 43.8% of patients in each cohort, therefore, the OS data was not mature yet. In the BM cohort, the percentage of PFS and iPFS event was 100.0 and 83.3%, respectively, and the median PFS was 2.3 months (95%CI 0.000–4.677), the median iPFS was 3.6 months (95%CI 1.435–5.705). In the LM cohort, the percentage of PFS and iPFS event was 56.3 and 56.3%, respectively, and the median PFS and the median iPFS was 4.3 months (95%CI 2.094–6.486). The analysis in survival was shown in Fig. 1 and Table 2.

**Table 2:** Clinical efficacy and improvements in CNS-related symptoms in the study population

	BM cohort (*N* = 12) N(%)	LM cohort (*N* = 16) N(%)
Median follow up duration		
Month (range)	6.3 (1.5–15.1)	7.8 (1.9–15.4)
Median iPFS, months (95%CI)	3.6 (1.435–5.705)	4.3 (2.094–6.486)
Median PFS, months (95%CI)	2.3 (0.000–4.677)	4.3 (2.094–6.486)
Overall best intracranial response		
ORR	2 (22.2)	–
DCR	8 (88.9)	–
CR	0 (0.0)	–
PR	2 (22.2)	–
SD	6 (66.7)	–
PD	1 (11.1)	–
Not evaluable	3 (25.0)	–
Overall best extracranial response		
ORR	1 (8.3)	1 (6.3)
DCR	4 (33.3)	7 (43.8)
CR	0 (0.0)	0 (0.0)
PR	1 (8.3)	1 (6.3)
SD	3 (25.0)	6 (37.5)
PD	4 (33.3)	1 (6.3)
Not evaluable	4 (33.3)	8 (50.0)
Number of OS events (%)	4 (33.3)	7 (43.8)
Number of PFS events (%)	12 (100.0)	9 (56.3)
Number of iPFS events (%)	10 (83.3)	9 (56.3)
Improvement in CNS-related symptoms		
Improvement	3 (100.0)	8 (53.3)
No improvement	0 (0.0)	4 (26.7)
Deterioration in symptoms	0 (0.0)	3 (20.0)

The percentages might not equal 100% on account of rounding

*n* number, *CNS* central nervous system, *iPFS* intracranial progression-free survival, *PFS* progression-free survival, *CR* complete remission, *PR* partial response, *SD* stable disease, *PD* progressive disease, *ORR* objective response rate (ORR = CR + PR), *DCR* disease control rate (DCR = CR + PR+ SD)

In the BM cohort, the best intracranial ORR was 22.2% (*n* = 2), with 0 CR (0.0%) and 2 PR (22.2%); CNS DCR was 88.9% (*n* = 8), with 0 CR (0.0%), 2 PR (22.2%), and 6 SD (66.7%) in 9 patients with at least one measurable lesion in brain (75.0%). The majority of patients in the LM cohort had non-target lesion, with only 6 patients (37.5%) accompanied with BM who had measurable lesions. Therefore, the efficacy in the LM cohort was only evaluated by PFS and iPFS in this study.

In 18 patients (64.3%) with CNS-related symptoms such as headache, fatigue, dizziness, and vomiting in all included patients, 61.1% (11/18) experienced improvement after the administration of furmonertinib with or without local therapy. Another 4 patients (22.2%) were reported to have at least no deterioration in symptoms after treatment, and only 3 patients (16.7%) had deterioration in symptoms. To be noticed, the majority of the LM cohort had CNS-related symptoms (15/16, 93.8%), and over a half of these patients had improvement from furmonertinib treatment (8/15, 53.3%).

### Subgroup analysis

**Fig. 2:**
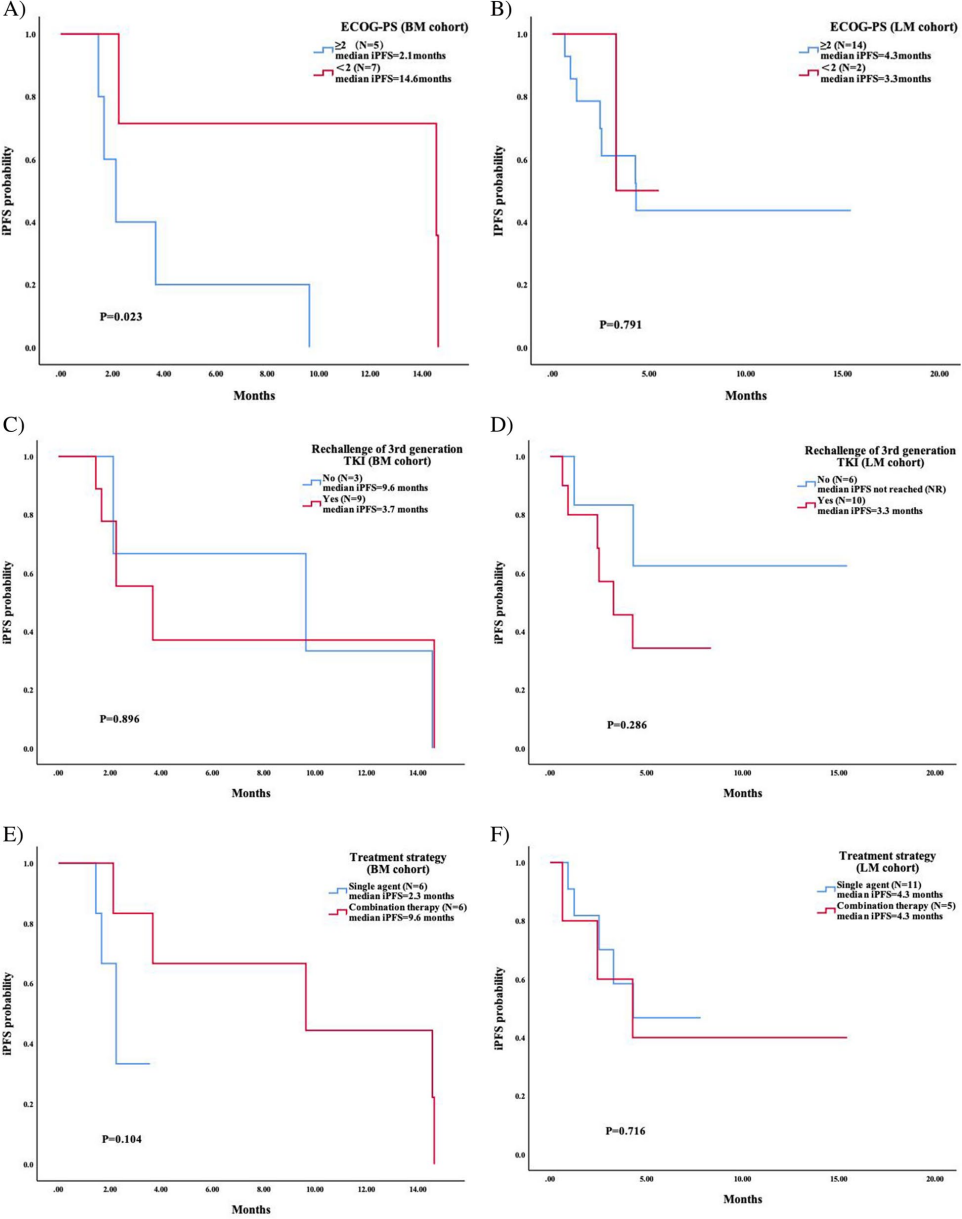
Survival analysis in each cohort with different characteristics and treatment strategies. Median intracranial progression-free survival (iPFS) in **A** the BM cohort, and **B** the LM cohort with different physical status. Median iPFS in **C** the BM cohort, and **D** the LM cohort who had treated with other third-generation TKI prior to furmonertinib or not. Median iPFS in **E** the BM cohort, and **F** the LM cohort treated with furmonertinib 160 mg monotherapy or in combination with anti-angiogenic agent

**Fig. 3:**
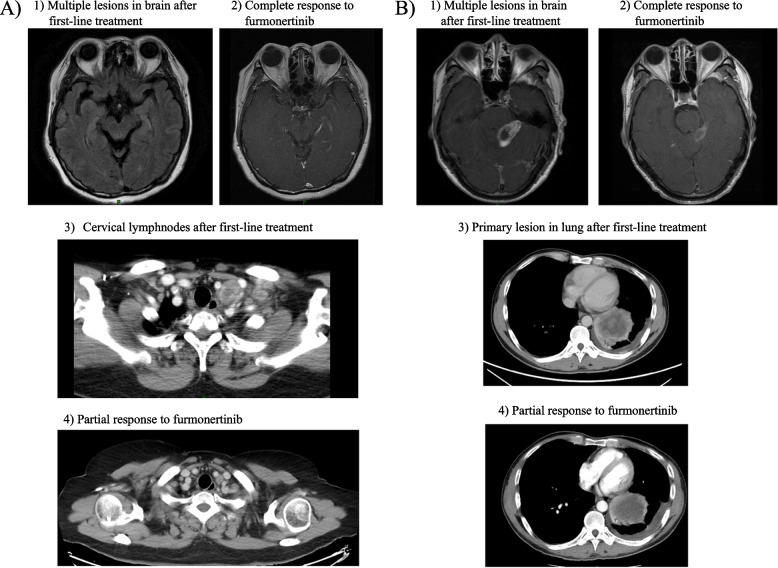
Typical examples in (epidermal growth factor receptor) *EGFR*-mutant non-small cell lung cancer (NSCLC) patients successfully treated with furmonertinib 160 mg as salvage treatment who had intracranial progression to prior tyrosine kinase inhibior (TKI). **A** A female patient had extracranial progression along with newly-diagnosed brain metastases (BM) and leptomeningeal metastases (LM) after first-line chemotherapy and afatinib. A secondary genetic test has shown *EGFR* exon 19del mutation (73.8%), *EGFR* exon20 T790M mutation (28.7%), and *EGFR* amplification (CN = 11.9). The patients then received furmonertinib 160 mg combining with bevacizumab as second-line treatment, and had a significant improvement in dizziness which was related to her central nervous system (CNS) disease. The targeted lesion in her brain had a complete response (CR), and the metastatic cervical lymph nodes also had a partial response (PR). 1) Multiple lesions in brain after first-line treatment; 2) Complete response to furmonertinib; 3) Cervical lymphnodes after first-line treatment; 4) Partial response to furmonertinib. **B** A male patient diagnosed as advanced *EGFR*-mutant NSCLC with BM received afatinib as first-line treatment for 14 months, and had an intracranial progression and edema with severe CNS-related symptoms such as fatigue and vomiting. A gene detection at progression showed *EGFR* exon19 deletion mutation (1.36%) and TP53 mutation (0.85%), whereas no T790M mutation in circulating tumor DNA (ctDNA). The patient then received furmonertinib 160 mg and bevacizumab along with radiotherapy in CNS. The targeted lesion in her brain and the primary lesion in lungs had a PR, and her symptoms were significantly relieved. 1) Multiple lesions in brain after first-line treatment; 2) Partial response to furmonertinib; 3) Primary lesion in lung after first-line treatment; 4) Partial response to furmonertinib

In the BM cohort, patients with an ECOG-PS of 0–1 achieved a significantly longer iPFS than those with a poor physical status (PS 2–3), which was 14.6 months (95%CI 0.000–32.594) and 2.1 months (95%CI 1.152–3.128), respectively (*P* = 0.023). In the LM cohort, the majority of patients (87.5%) had a poor physical status prior to furmonertinib treatment, therefore no significant difference was observed between patients with different status (*P* = 0.791). In addition, the survival analysis in patients who had received third-generation TKI rechallenge (including patients with or without other treatments in between) seemed to have an inferior iPFS compared to those who had not, while no significant difference was observed in each cohort. As for treatment strategies after CNS progression to prior TKI, the combination of furmonertinib 160 mg and bevacizumab/anlotinib has improved the iPFS compared to single agent in the BM cohort, with the median iPFS of 9.6 months (95%CI 0.000–21.322) and 2.3 months (95%CI 1.605–2.895), respectively, although no significant significance was observed (*P* = 0.104). However, no similar trend was observed in the LM cohort (*P* = 0.903). The subgroup analysis above was shown in Fig. 2. Two typical cases in the BM cohort and the LM cohort who were successfully treated with furmonertinib 160 mg and bevacizumab were presented in Fig. 3, and the treatment strategies for patients who had adchived an iPFS of more than 6 months were presented in Supplementary Table 1.

The hazard ratio of iPFS in the BM cohort with different characteristics using univariate analysis was shown in Supplementary Fig. 1. Patients with an ECOG-PS ≥2 had a significantly inferior iPFS than those < 2 (HR = 5.503, 95%CI 1.046–28.965, *P* = 0.044). Other factors have shown no significant impact. The hazard ratio of iPFS in the LM cohort with different characteristics using univariate COX proportional hazards regression model was shown in Supplementary Fig. 2. Patients who had received less than 2 lines of previous systemic treatment achieved a significantly longer iPFS than those who had received more than 1 line (HR = 0.060, 95%CI 0.006–0.562, *P* = 0.014). Other factors such as the concurrent progression in BM and the concurrent application of intrathecal injection have shown no great impact to iPFS.

### Safety profile of Furmonertinib as salvage treatment

Overall, any grade of all-cause adverse events (AEs) occured in 46.4% of all included patients (13 of 28) who received furmonertinib 160 mg with or without bevacizuamb or anlotinib as subsequent therapy, as recorded in Table 3. The most common AEs were decreased blood cell count (25.0%), increased alanine aminotransferase (ALT) and aspartate aminotransferase (AST) (17.9%), and decreased appetite (10.7%). Among them, 14.3% of patients (4 of 28) had grade 3 or higher AEs, including 1 case of grade 4 decreased blood cell count, 1 case of grade 3 increased ALT/AST, 1 case of grade 3 fatigue and decreased appetite, and 1 case of grade 3 hypocalcaemia. Notably, no patients have suspended or discontinued furmonertinib due to any AEs, even those who received combination therapy of furmonertinib and chemotherapy. Also, no dose reductions had occurred owing to treatment-related AEs, and no AEs were related to death.

**Table 3:** Overview of AEs in all patients who received furmonertinib 160 mg monotherapy or combination therapy as subsequent treatment

Adverse events	Grade 1	Grade 2	Grade 3	Grade 4
	n (%)	n (%)	n (%)	n (%)
General Disorders				
Fatigue	2 (7.1)		1 (3.6)	
Decreased appetite	2 (7.1)		1 (3.6)	
Hematologic toxicity				
Leukopenia	3 (10.7)	1 (3.6)		1 (3.6)
Anemia	5 (17.9)	2 (7.1)		
Decrased platelet count				1 (3.6)
Gastrointestinal toxicity				
Mucositis oral			1 (3.6)	
Diarrhea		1 (3.6)		
Renal toxicity				
Increased serum creatinine		2 (7.1)		
Metabolism disorders				
Hypocalcemia			1 (3.6)	
Hepatobiliary toxicity				
Increased ALT/AST	4 (14.3)		1 (3.6)	

*n* number, *AEs* adverse events, *ALT* alanine aminotransferase, *AST* aspartate aminotransferase

## Discussion

The occurrence of CNS progression in the course of *EGFR*-mutated NSCLC predicts poor outcomes, and requires an optimal theraeutic strategy. Moreover, a large number of patients who had received multiple lines of systemic treatments could only receive best supportive care (BSC), owing to the weak physical status, and had little improval in survival. Therefore we designed this retrospective study to evaluated the efficacy of furmonertinib in NSCLC patients who had developed BM/LM progression from previous TKI treatment. With more than a half of the included patients having a poor physical status and CNS-related symptoms, the administration of single-agent furmonertinib 160 mg or in combination of anti-angiogenic agents has achieved a median iPFS of 3.6 months (95%CI 1.435–5.705) in the BM cohort, and 4.3 months (95%CI 2.094–6.486) in the LM cohort. The intracranial ORR and DCR reached 16.7 and 66.7% in the BM cohort. Subgroup analysis and univariate analysis has shown that a good ECOG-PS correlated with a favorable efficacy of furmonertinib in the BM cohort. The AEs were all under control, and led to no dose reductions or suspension. Compared to historic data in NSCLC with BM receiving BSC only, which achieved an average survival of merely about 3 months [21], our results have implied a novel therapeutic strategy for advanced NSCLC patients with BM/LM progression from previous TKI treatment. On the basis of this study, we have conducted an open-label, prospective phase II trial (iFORCE, NCT05465343) to bring more evidence of the applying furmonertinib 160 mg in selected patients who progressed in CNS from prior TKI, and the trial is still recruiting.

Treatment strategies for symptomatic CNS disease in NSCLC are limited, and are still under investigation. Localized treatments such as radiotherapy and intrathecal injection with chemotherapy help to release CNS-related symptoms in BM/LM, while are also controversial in prolonging long-term survival. Concurrent use of systemic treatment is still required for disease control. Cytotoxic agents in chemotherapy are poor in penetrating the BBB, and therefore provide limited efficacy for BM [22]. Albeit an improved response and a lower cumulative risk of CNS progression for first-generation TKI compared to chemotherapy [23], first- and second-generation TKIs distributed poorly in the brain, partly owing to the interaction with permeability glycoprotein (P-gp) and breast cancer-resistance protein (BCRP) [24]. Preclinical data has shown that third-generation TKI osimertinib had an improved brain exposure than rociletinib and afatinib, with a higher concentration and brain/plasma concentration ratio [25]. In the randomized phase 3 trial FLAURA study, osimertinib was demonstrated to have a significantly longer intracranial PFS than gefitinib in the first-line setting (HR 0.47, 95%CI 0.30–0.74; *p* = 0.007) [6]. In *EGFR* T790M-positive patients who progressed from previous TKI with BM or LM, a double dose of osimertinib (160 mg) has provided promising intracranial response rates and survival benefit [16]. The BLOOM study has shown an ORR of 62% and a DoR of 15.2 month in patients with LM regardless of T790M status [17]. The preclinical and clinical evidence has facilitated the use of third-generation targeted agent osimertinib.

Another third-generation TKI furmonertinib also has the promising efficacy in treating CNS diseases in NSCLC. Preclinical studies have demonstrated the BBB penetration ability of furmonertinib, which allowed it to inhibit brain and spinal cord metastases in NSCLC [26]. The phase 2a study has shown a promising clinical efficacy of furmonertinib in *EGFR* T790M-mutated NSCLC with CNS metastases, especially in the 160 mg group [18]. The phase 2b study has further explored the efficacy of furmonertinib in T790M-positive NSCLC, including 48% of patients with asymptomatic stable CNS metastases at baseline [19]. Results have shown that the CNS ORR and DCR were 66 and 100%, respectively, and the intracranial mPFS was 11.6 months (95% CI 8.3–13.8) [19]. These studies have indicated a robust CNS activity of furmonertinib in patients with T790M resistance mutation. The FURLONG study has also included patients with asymptomatic stable CNS metastases with sensitizing *EGFR* mutations, and has reported a superior CNS mPFS of furmonertinib over gefitinib (20.8 vs. 9.8 months, HR = 0.40, 95%CI 0.23, 0.71, *p* = 0.0011) in 133 patients with CNS lesions (37% of 358 patients from the FURLONG study) [27]. Similar to osimertinib, double dose of furmonertinib has achieved a relatively promoted response and efficacy in patients with BM (CNS ORR being 60.0% with 80 mg Qd and 84.6% with 160 mg Qd; CNS PFS being 9.7 months with 80 mg Qd and 19.3 months with 160 mg Qd) [28]. Based on the evidence from previous studies, a dose of 160 mg was adopted in our study, and has also achieved a promising result in survival.

Despite a promoted PFS and response rate of osimertinib compared to first-generation TKI and chemotherapy, the resistance to osimertinib has been more complexed, including on-target and off-target acquired mechanisms [29]. Therefore, the optimal therapeutic strategies following osimertinib in *EGFR*-mutated NSCLC patients are emerging, guided by molecular tests. For example, the frequently-detected resistance mechanism *MET* amplification and *HER2* amplification could be targeted via the combination of crizotinib [30] or pyrotinib [31]. The detection for resistance mechanisms is recommended by tissue rebiopsy or by liquid biopsy, whereas the former assay is less practical especially after previous anti-tumor therapy, owing to the insufficient sample for genetic analysis. Liquid biopsy via circulating tumor DNA (ctDNA) has developed with its high accordance to tissue testing in determining *EGFR* status [32]. However, peripheral ctDNA may not indicate CNS progression as efficiently as it predicts extracranial metastases [33], and thus CSF ctDNA is more recommended for CNS malignancies [34]. A relatively large proportion of each group had a negative or unknown T790M status prior to furmonertinib, probably attributing to the false-negative genetic results via peripheral blood, has limited our exploration of the underlying mechanism in this study. Besides, the genetic examination was done in only several cases in the LM cohort, which provided scarce information. Future studies with larger sample for gene detection are needed to characterize the CNS diseases and to facilitate the clinical management. Daoan Cheng et al. has reported a case of an advanced NSCLC patient who progressed from second-line osimertinib, manifested as diffused brain and lung metastases, with the loss of T790M mutation and exon 19 deletion, benefited from furmonertinib at a dose of 160 mg Qd as salvage therapy [35]. Although the report failed to suggest a possible mechanism, it did indicate a potential treatment option for osimertinib-resistant patients. In our study, the rechallenge of furmonertinib was less likely to benefit in survival compared to those who had not received osimertinib prior to furmonertinib (median iPFS of 3.7 months vs. 9.6 months in the BM cohort, and of 3.3 months vs. NR in the LM cohort). However, no significant difference was observed, and several cases did achieve a noticeable response to furmonertinib rechallenge after CNS progression to osimertinib. Therefore, the feasibility of furmonertinib rechallenge and the underlying mechanisms are worth of exploration.

To our knowledge, this is the first real-world study of double-dose furmonertinib in advanced NSCLC patients who progressed in BM/LM from previous *EGFR*-TKI, partly heavily-treated and physically weak. There are still several limitations in our study. First, as a retrospective study, the sample size is relatively small, especially for subgroup analysis. A trend of favorable iPFS was observed in selected patients who had not received osimertinib prior to furmonertinib, and in patients who received furmonertinib combination therapy, however, no significant difference was found due to the limitation of sample size. Therefore, further study is still required to explore the factors that impact the benefit from the treatment strategy of furmonertinib monotherapy or combination therapy after BM/LM progression. Second, the detailed information regarding radiotherapy is lacking, which limits the exploration of the correlation between RT and efficacy in BM/LM population. Third, since a proportion of patients had denied further gene tests, our study failed to reveal the underlying mechanism of patients with BM/LM who benefited from the treatment strategy. It remains to be explored to guide precision and personalized medicine in the future prospective studies with large sample.

## Conclusion

Single-agent furmonertinib 160 mg or in combination of anti-angiogenic agent is an optional salvage therapy for advanced NSCLC patients who developed BM/LM progression from prior *EGFR*-TKI treatment, with a promising efficacy and an acceptable safety profile, and is worth of further exploration.

## Supplementary Information

**Additional file 1: Supplementary Table 1. **Treatment history and strategies for patients who achieved a long duration of intracranial response after furmonertinib 160mg with or without anti-angiogenic agent as salvage therapy.

**Additional file 2: Supplementary Figure 1. **Hazard ratio of PFS in the BM cohort with different characteristics who received furmonertinib 160mg with or without anti-angiogenic agent as salvage therapy using univariate analysis. A hazard ratio less than 1 implies a lower risk of disease progression or death in group 1 than in group 2.** Supplementary Figure 2. **Hazard ratio of PFS in the LM cohort with different characteristics who received furmonertinib 160mg with or without anti-angiogenic agent as salvage therapy using univariate analysis. A hazard ratio less than 1 implies a lower risk of disease progression or death in group 1 than in group 2.

## Data Availability

The datasets used and/or analyzed during the current study available from the corresponding author on reasonable request.

## References

[1] Sung H, Ferlay J, Siegel RL (2021). Global Cancer statistics 2020: GLOBOCAN estimates of incidence and mortality worldwide for 36 cancers in 185 countries. CA Cancer J Clin.

[2] Lynch TJ, Bell DW, Sordella R (2004). Activating mutations in the epidermal growth factor receptor underlying responsiveness of non-small-cell lung cancer to gefitinib. N Engl J Med.

[3] Kwak EL, Bang YJ, Camidge DR (2010). Anaplastic lymphoma kinase inhibition in non-small-cell lung cancer. N Engl J Med.

[4] Maemondo M, Inoue A, Kobayashi K (2010). Gefitinib or chemotherapy for non-small-cell lung cancer with mutated EGFR. N Engl J Med.

[5] Wu YL, Zhou C, Hu CP (2014). Afatinib versus cisplatin plus gemcitabine for first-line treatment of Asian patients with advanced non-small-cell lung cancer harbouring EGFR mutations (LUX-lung 6): an open-label, randomised phase 3 trial. Lancet Oncol.

[6] Soria JC, Ohe Y, Vansteenkiste J (2018). Osimertinib in untreated EGFR-mutated advanced non-small-cell lung Cancer. N Engl J Med.

[7] Lu S, Dong X, Jian H, et al. AENEAS: a randomized phase III trial of Aumolertinib versus Gefitinib as first-line therapy for locally advanced or metastatic non-small-cell lung Cancer with EGFR exon 19 deletion or L858R mutations. Journal of clinical oncology: official journal of the American society of. Clin Oncol. 2022;Jco2102641:3162–71. 10.1200/JCO.21.02641 PMC9509093 35580297

[8] Mok TS, Wu YL, Ahn MJ (2017). Osimertinib or platinum-Pemetrexed in EGFR T790M-positive lung Cancer. N Engl J Med.

[9] Lu S, Wang Q, Zhang G (2022). Efficacy of Aumolertinib (HS-10296) in patients with advanced EGFR T790M+ NSCLC: updated post-National Medical Products Administration Approval Results from the APOLLO Registrational trial. J Thoracic Oncol.

[10] Kobayashi S, Boggon TJ, Dayaram T (2005). EGFR mutation and resistance of non-small-cell lung cancer to gefitinib. N Engl J Med.

[11] Ernani V, Stinchcombe TE (2019). Management of Brain Metastases in non-small-cell lung Cancer. J Oncol Pract.

[12] Lee J, Ahn MJ (2021). Brain metastases in patients with oncogenic-driven non-small cell lung cancer: pros and cons for early radiotherapy. Cancer Treat Rev.

[13] Li YS, Jiang BY, Yang JJ (2016). Leptomeningeal metastases in patients with NSCLC with EGFR mutations. J Thoracic Oncol.

[14] Umemura S, Tsubouchi K, Yoshioka H (2012). Clinical outcome in patients with leptomeningeal metastasis from non-small cell lung cancer: Okayama lung Cancer study group. Lung Cancer (Amsterdam, Netherlands).

[15] Xu Y, Hu M, Zhang M (2018). Prospective study revealed prognostic significance of responses in leptomeningeal metastasis and clinical value of cerebrospinal fluid-based liquid biopsy. Lung Cancer (Amsterdam, Netherlands).

[16] Park S, Lee MH, Seong M (2020). A phase II, multicenter, two cohort study of 160 mg osimertinib in EGFR T790M-positive non-small-cell lung cancer patients with brain metastases or leptomeningeal disease who progressed on prior EGFR TKI therapy. Ann Oncol.

[17] Yang JCH, Kim SW, Kim DW (2020). Osimertinib in patients with epidermal growth factor receptor mutation-positive non-small-cell lung Cancer and leptomeningeal metastases: the BLOOM study. J Clin Oncol.

[18] Shi Y, Zhang S, Hu X (2020). Safety, clinical activity, and pharmacokinetics of Alflutinib (AST2818) in patients with advanced NSCLC with EGFR T790M mutation. J Thoracic Oncol.

[19] Shi Y, Hu X, Zhang S (2021). Efficacy, safety, and genetic analysis of furmonertinib (AST2818) in patients with EGFR T790M mutated non-small-cell lung cancer: a phase 2b, multicentre, single-arm, open-label study. Lancet Respir Med.

[20] Shi Y, Chen G, Wang X, Liu Y, Wu L, Hao Y (2022). Furmonertinib (AST2818) versus gefitinib as first-line therapy for Chinese patients with locally advanced or metastatic EGFR mutation-positive non-small-cell lung cancer (FURLONG): a multicentre, double-blind, randomised phase 3 study. Lancet Respir Med.

[21] Borgelt B, Gelber R, Kramer S (1980). The palliation of brain metastases: final results of the first two studies by the radiation therapy oncology group. Int J Radiat Oncol Biol Phys.

[22] Bernardo G, Cuzzoni Q, Strada MR (2002). First-line chemotherapy with vinorelbine, gemcitabine, and carboplatin in the treatment of brain metastases from non-small-cell lung cancer: a phase II study. Cancer Investig.

[23] Heon S, Yeap BY, Lindeman NI (2012). The impact of initial gefitinib or erlotinib versus chemotherapy on central nervous system progression in advanced non-small cell lung cancer with EGFR mutations. Clin Cancer Res.

[24] de Vries NA, Buckle T, Zhao J, Beijnen JH, Schellens JH, van Tellingen O (2012). Restricted brain penetration of the tyrosine kinase inhibitor erlotinib due to the drug transporters P-gp and BCRP. Investig New Drugs.

[25] Ballard P, Yates JW, Yang Z (2016). Preclinical comparison of Osimertinib with other EGFR-TKIs in EGFR-mutant NSCLC brain metastases models, and early evidence of clinical brain metastases activity. Clin Cancer Res.

[26] Zhang Y, Zhang Y, Niu W (2021). Experimental study of Almonertinib crossing the blood-brain barrier in EGFR-mutant NSCLC brain metastasis and spinal cord metastasis models. Front Pharmacol.

[27] Shi Y, Chen G, Wang X, Liu Y, Wu L, Hao Y, et al. Central nervous system efficacy of furmonertinib (AST2818) versus gefitinib as first-line treatment for EGFR-mutated NSCLC: results from the FURLONG study. J Thorac Oncol. 2022:S1556-0864(22)01496-4. 10.1016/j.jtho.2022.07.1143. Epub ahead of print. 10.1016/j.jtho.2022.07.1143 35932953

[28] Shi Y, Hu X, Liao W (2021). P76. 65 CNS efficacy of AST2818 in patients with T790M-positive advanced NSCLC: data from a phase I-II dose-expansion study. J Thorac Oncol.

[29] Zeng Y, Yu D, Tian W, Wu F (2022). Resistance mechanisms to osimertinib and emerging therapeutic strategies in nonsmall cell lung cancer. Curr Opin Oncol.

[30] Blasi M, Kazdal D, Thomas M (2021). Combination of Crizotinib and Osimertinib in T790M+ EGFR-mutant non-small cell lung Cancer with emerging MET amplification post-Osimertinib progression in a 10-year survivor: a case report. Case Rep Oncol.

[31] Gan J, Huang Y, Liao J, Pang L, Fang W (2021). HER2 amplification in advanced NSCLC patients after progression on EGFR-TKI and clinical response to EGFR-TKI plus Pyrotinib combination therapy. OncoTargets Therapy.

[32] Douillard JY, Ostoros G, Cobo M (2014). Gefitinib treatment in EGFR mutated caucasian NSCLC: circulating-free tumor DNA as a surrogate for determination of EGFR status. J Thoracic Oncol.

[33] Garcia-Murillas I, Chopra N, Comino-Méndez I (2019). Assessment of molecular relapse detection in early-stage breast Cancer. JAMA Oncol.

[34] Escudero L, Martínez-Ricarte F, Seoane J (2020). Cerebrospinal fluid circulating tumour DNA as a liquid biopsy for central nervous system malignancies. Curr Opin Neurol.

[35] Cheng D, Tang S, Li D (2022). Successful salvage therapy using high-dose furmonertinib (AST2818) for non-small-cell lung cancer after Osimertinib resistance: a case report. Anti-Cancer Drugs.

