# Supplementary material for: Intracranial efficacy and safety of furmonertinib 160 mg with or without anti-angiogenic agent in advanced NSCLC patients with BM/LM as salvage therapy
Source: BMC Cancer. 2023 Mar 4;23:206. doi: 10.1186/s12885-023-10676-x (PMC9985196; doi:10.1186/s12885-023-10676-x)

**Supplementary Figure 1.** Hazard ratio of PFS in the BM cohort with different characteristics who received furmonertinib 160mg with or without anti-angiogenic agent as salvage therapy using univariate analysis. A hazard ratio less than 1 implies a lower risk of disease progression or death in group 1 than in group 2.


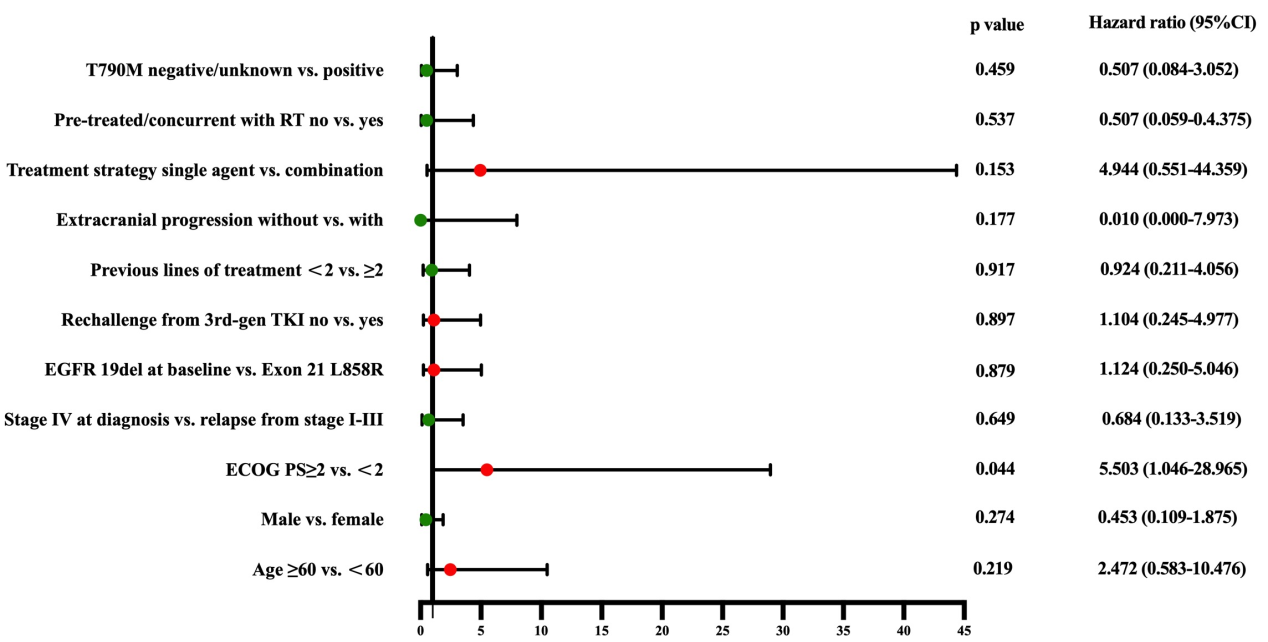


**Supplementary Figure 2.** Hazard ratio of PFS in the LM cohort with different characteristics who received furmonertinib 160mg with or without anti-angiogenic agent as salvage therapy using univariate analysis. A hazard ratio less than 1 implies a lower risk of disease progression or death in group 1 than in group 2.


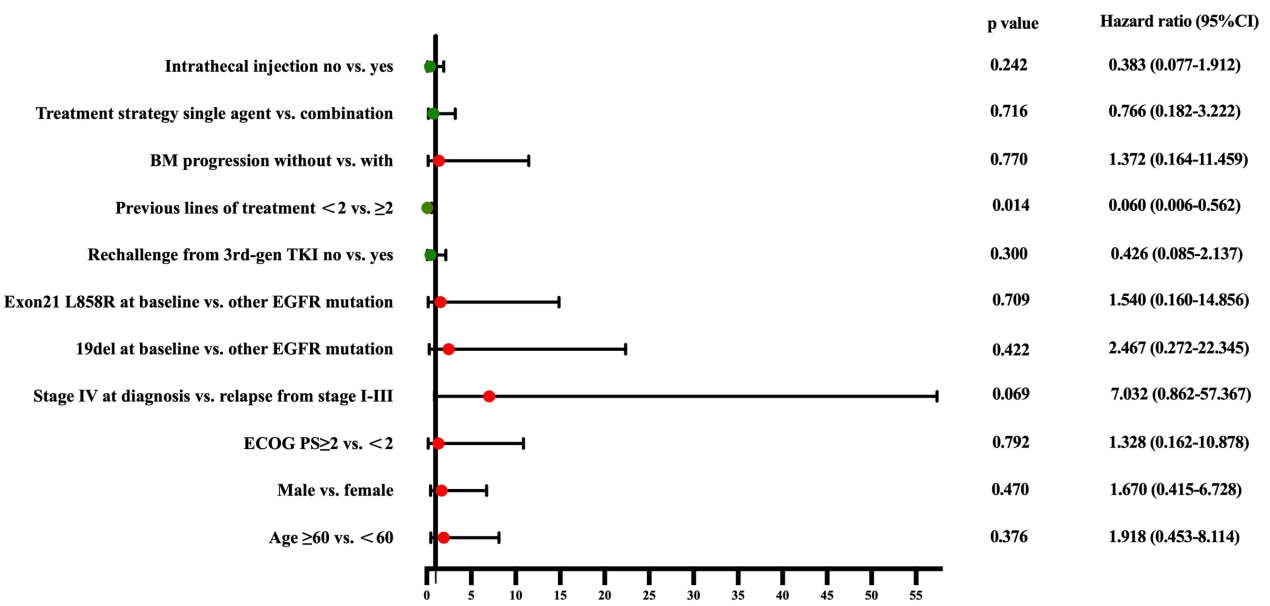

Supplement: Supplementary file 2 — Additional file 2: Supplementary Figure 1. Hazard ratio of PFS in the BM cohort with different characteristics who received furmonertinib 160mg with or without anti-angiogenic agent as salvage therapy using univariate analysis. A hazard ratio less than 1 implies a lower risk of disease progression or death in group 1 than in group 2. Supplementary Figure 2. Hazard ratio of PFS in the LM cohort with different characteristics who received furmonertinib 160mg with or without anti-angiogenic agent as salvage therapy using univariate analysis. A hazard ratio less than 1 implies a lower risk of disease progression or death in group 1 than in group 2. [file 12885_2023_10676_MOESM2_ESM.docx]
